# Maximum Recommended Dosage of Lithium for Pregnant Women Based on a PBPK Model for Lithium Absorption

**DOI:** 10.1155/2012/352729

**Published:** 2012-05-30

**Authors:** Scott Horton, Amalie Tuerk, Daniel Cook, Jiadi Cook, Prasad Dhurjati

**Affiliations:** ^1^Colburn Laboratory, Department of Chemical and Biomolecular Engineering, University of Delaware, Newark, DE 19716, USA; ^2^Resident in the Department of Family Medicine, Christiana Care Health Services, Wilmington, DE 19805, USA

## Abstract

Treatment of bipolar disorder with lithium therapy during pregnancy is a medical challenge. Bipolar disorder is more prevalent in women and its onset is often concurrent with peak reproductive age. Treatment typically involves administration of the element lithium, which has been classified as a class D drug (legal to use during pregnancy, but may cause birth defects) and is one of only thirty known teratogenic drugs. There is no clear recommendation in the literature on the maximum acceptable dosage regimen for pregnant, bipolar women. We recommend a maximum dosage regimen based on a physiologically based pharmacokinetic (PBPK) model. The model simulates the concentration of lithium in the organs and tissues of a pregnant woman and her fetus. First, we modeled time-dependent lithium concentration profiles resulting from lithium therapy known to have caused birth defects. Next, we identified maximum and average fetal lithium concentrations during treatment. Then, we developed a lithium therapy regimen to maximize the concentration of lithium in the mother's brain, while maintaining the fetal concentration low enough to reduce the risk of birth defects. This maximum dosage regimen suggested by the model was 400 mg lithium three times per day.

## 1. Introduction

Bipolar disorder, which affects approximately 1% of the population (mostly women), is a type of mood disorder which has periods of manic behavior and periods of depressive behavior. An overly joyful or overexcited state characterizes manic behavior; extremely sad and hopeless states characterize depressive behavior [1]. A standard treatment for bipolar disorder involves treatment with the element lithium, which was the first mood-stabilizing medication approved for treatment of “mania,” which later came to be known as bipolar disorder, in 1970 [2]. The brand names of bipolar lithium treatment drugs are Eskalith and Lithobid, which deliver lithium as lithium carbonate (Li_2_CO_3_). The typical size of a dose of lithium drug ranges from 900 to 1800 mg Li_2_CO_3_/day (if administered in 2 divided doses a day) and 900 to 2400 mg Li_2_CO_3_/day (if administered in 3-4 divided doses a day) [3]. Although the mechanism by which lithium mitigates the symptoms of bipolar disorder is not completely understood, lithium is thought to affect sodium transfer in the brain [4]. High doses can cause lithium poisoning and side effects such as the inability to control movement, blackouts, seizures, hallucinations, severe headaches, and acute renal failure [3, 5].

In women, bipolar disorder typically manifests prior to the age of 30, which coincides with the timing of a woman's peak reproduction age [4]. Unfortunately, lithium is also classified as one of only thirty known teratogenic drugs (i.e., substances associated with causing birth defects) [6]. Therefore, the treatment of pregnant women for bipolar disorder with lithium presents a major risk for the safety of the fetus. Lithium crosses the human placenta freely [2] and affects vasculature formation in the fetus [4]. Because the majority of vasculature forms during the first trimester, lithium affects the development of the fetus most in the first trimester [7]. The most common birth defects associated with 30 babies exposed to lithium during pregnancy were hypotonia (43%); cardiac problems (40%); respiratory distress syndrome and cyanosis (30%); poor feeding ability, lethargy, and depressed Moro and suck reflexes (27%) [2].

Although lithium treatment concurrent with pregnancy has the potential to cause serious birth defects, there is little guidance toward appropriate lithium treatment regimens for pregnant women. Current FDA-approved packaging for Lithobid states, “If this drug is used in women of childbearing potential, or during pregnancy, or if a patient becomes pregnant while taking this drug, the patient should be apprised by their physician of the potential hazard to the fetus” [8]. The extent of the current guidance by physicians is to avoid lithium if possible during the first trimester of pregnancy; if this is not possible, and during the second and third trimesters, the lowest effective dose of lithium should be used [3].

It would be beneficial to quantitatively establish a maximum acceptable dosage regimen that will not cause teratogenic effects on the fetus. However, measuring the actual concentration in the fetus is prohibitively difficult and has a potential to cause damage to the fetus. Furthermore, a large dataset associating actually administered lithium therapy regimens correlated with instances and noninstances of birth defects would be required in order to conclusively establish a correlation of lithium exposure with birth defect incidence; this data is not currently available. Additionally, animal models describing fetal lithium toxicity or teratogenic effects are lacking.

In lieu of these experimental methods, lithium concentration in various organs can be predicted by the construction of a pharmacokinetic model, such as that presented by Bischoff et al. [9]; this provides a means to predict lithium concentration within the fetus during various dosage regimens based upon the concentrations in other parts of the mother's body which are easier and safer to sample. By modeling the effect of dosage regimens previously associated with high incidence of birth defects, improved dosage regimens with lower likelihoods of causing birth defects can be proposed. To this end, we have employed a modified version of the biological model first proposed by Bischoff et al. [9] (the Physiologically Based Pharmacokinetic (PBPK) model). PBPK models have been developed and validated for a variety of applications since they were first introduced, including predicting organophosphate insecticide concentration in humans [10], modeling toxicology of complex mixtures [11], and drug discovery [12]. This work applies a modified PBPK model to the problem of impaired fetal development due to maternal lithium treatment in order to propose maximum recommended dosage regimens.

## 2. Basis and Development of Model

### 2.1. General Assumptions of the Model

A few assumptions and features of this model warrant discussion. First, this mass-transfer-based model includes organ “compartments”; each compartment represents a “lumped” region of organs/tissues with similar physiochemical properties. All properties of the “compartment” (volume, blood flow rate, etc.) have independently verifiable anatomical significance [13]. Another assumption employed in the model development was an assumption of flow-limited conditions; this implies that the blood leaving a tissue/organ is in diffusion equilibrium with the tissue/organ, such that the perfusion rate is rate controlling rather than the diffusion rate. The rationale and justification for this assumption is discussed in detail in Bischoff et al. [13]. Finally, in this model, each compartment is modeled as a continuously stirred tank reactor (CSTR), with constant volume, constant inflow and outflow rates, and a blood permeable partitioning membrane through which lithium enters the organ/tissue along with the blood. Each organ/tissue has a specific membrane partition constant to characterize the relative partitioning of the drug between the blood, which delivers the drug, and the tissue; this parameter can also be described as the tissue to plasma equilibrium distribution ratio. These values are contained in Table 1. These distribution ratios were found from published literature for all compartments except the fetus, for which data was not available. In addition, there is no published literature that provides absolute fetal lithium concentrations associated with lithium therapy regimens. However, dosage regimens which have resulted in birth defects have been documented [2]. Without a correlation of fetal concentrations to these known pathological dosage regimens, the value of the fetal compartment partition coefficient (*R*_F_) can be chosen arbitrarily; this allowed us to explore the effect of different lithium therapy regimens on relative fetal concentration. Since the fetal lithium concentrations reported by the simulation are relative to pathological cases, any value chosen for *R*_F_ will simply give scaled results leading to the same conclusions. This makes selecting a particular value for *R*_F_ arbitrary. We selected a value of 0.8 as the fetal partition coefficient to use in our model.

**Figure 1:**
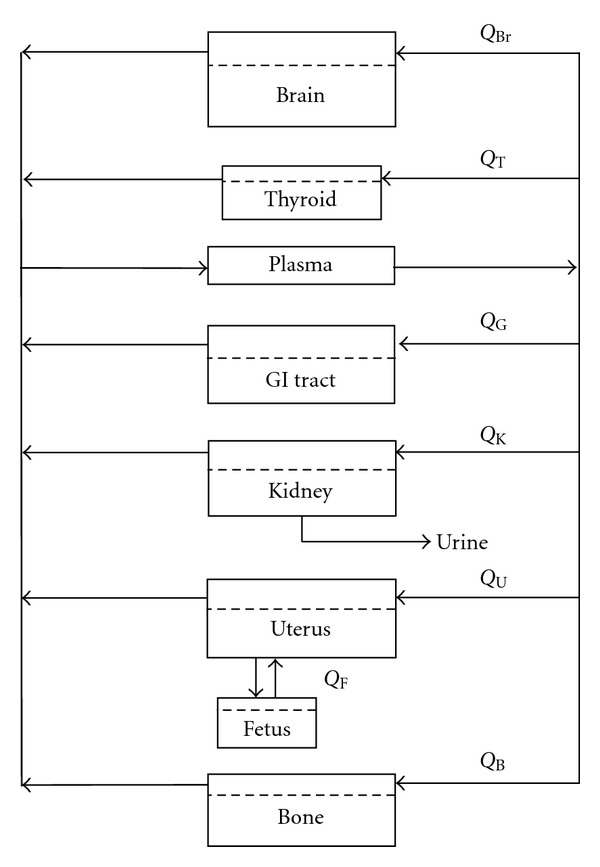
Connectivity diagram for the relevant organs of the PBPK model for lithium accumulation in a pregnant woman. Each compartment represents an organ with a certain partition coefficient, denoted by a dashed line, through which blood flows.

The original model of Bischoff et al. [9] predicted concentrations of the anticancer drug methotrexate in different organs in the body. Because methotrexate is metabolized in the liver and secreted through the colon without a concentration buildup in the upper organs, Bischoff et al. [9] modeled only the lower organs in the body. Our model, however, deals with lithium, for which the brain is a primary site of lithium activity and accumulation. Furthermore, as we are specifically modeling the lithium exposure of pregnant women, the fetus and the uterus are additional physiological regions for which lithium concentrations should be predicted. Therefore, we have modified the original PBPK model to better reflect our system by adding compartments for the brain, the uterus, and the fetus, and modifying the compartment connectivity accordingly (see Figure 1). In our model, we assumed that the fetus was in the first trimester, when lithium exposure is most damaging to its development. Due to the lower development of the fetus at this point in the pregnancy, we modeled the fetus as a single compartment. Our model also included thyroid, gastrointestinal (GI) tract, kidneys, and bone compartments.

### 2.2. Physiological Considerations of the Model

In order to investigate the effect of lithium dosage regimens on maternal organ and fetal concentrations, data on lithium partitioning between blood plasma and the relevant organs and tissues is required. This enables the model to predict which organs/tissues preferentially uptake and concentrate lithium. We obtained partitioning coefficients directly from the medical database Lexi-Comp Online [3] for all organs except the fetus, for which data was not available. The kidneys are the main source of lithium clearance from the body; approximately 99% of the lithium in the body is removed through the urine, while the remaining 1% passes through feces. Therefore this model assumes that the lithium clearance through the feces is negligible and only considers kidney clearance for lithium removal. The GI tract is included because an oral mode of delivery is employed for lithium-containing bipolar drugs, thus lithium is ingested and absorbed through the GI tract.

Lexi-Comp Online indicated that lithium accumulates in the brain, thyroid, and bone tissue. Therefore, these organs have the possibility to significantly affect the concentrations in important organs by absorbing a majority of lithium when blood plasma concentrations are high or releasing lithium even when blood plasma concentrations are low.

The connectivity of the model, as shown in Figure 1, is based on clinical physiology. Each compartment represents an organ with a certain partition coefficient, denoted by a dashed line, through which blood flows. The box marked “Plasma” can be viewed as the aorta, or the main pathway for blood flow through the body; within the aorta, mixing of the blood/plasma is assumed to occur so that the blood exiting this compartment, destined to the other organs of the body, has a single concentration at any point in time (*C*_P_). The plasma splits into two pathways; the upper pathway proceeds to the thyroid and brain, while the lower pathway proceeds to the GI tract, kidneys, uterus, and bone. As in the body, blood flows through these organs in parallel. Since the fetus is connected to the mother through the uterus, the flow of blood to and from the fetus travels through the uterus. According to Kozma [2], lithium crosses the human placenta freely, and the concentrations in fetal serum are equal to that of maternal serum; this enables us to assume that the concentration of lithium in the blood passing entering the fetus has the same concentration as that of the blood leaving the uterus. Because our model assumes a fetus in the first trimester, the renal system was assumed to be immature such that renal clearance of lithium from the fetus would be negligible [2]. Therefore the only route for lithium to exit the fetus is partitioning back from the fetus to the plasma. This has the additional effect of causing a delayed clearance of lithium from the fetus (and accumulation of lithium within the fetus), so that toxic levels of lithium may be experienced by the fetus even though the concentration of lithium for the mother may be within the therapeutic range [2].

### 2.3. Mathematical Considerations of the Model-Coupled Differential Equations

The following coupled differential equations describe the time dependence of lithium concentrations in the compartments from Figure 1. Based upon a general mass balance, the rate of accumulation of lithium ((*d*/*dt*)(*V*_*i*_ *C*_*i*_)) within the control volume (*V*_*i*_) of a general physiological compartment *i* is equal to the rate of lithium entering the compartment minus the rate of lithium leaving the compartment minus the rate of lithium clearance from the system plus the rate of lithium absorption to the system, as shown in (1).

**General organ/tissue mass balance:**

(1) $$\begin{array}{c} \frac{d}{dt}\;\left(V_{i}C_{i}\right)=r_{Li,\text{entering}}-r_{Li,\text{exiting}}-r_{Li,\text{clearance}}+r_{Li,\text{absorption}} \end{array}$$

For a general compartment *i* (exceptions are discussed below), the rate of lithium entering the compartment is the product of the plasma flow rate to the compartment (*Q*_*i*_) and the concentration of lithium in the plasma (*C*_P_); the rate of lithium leaving a general compartment is the product of the plasma flow out of the compartment (which is equivalent to the flow in, *Q*_*i*_) and the concentration of lithium in the blood flowing out. The concentration of lithium in the blood exiting an organ will be a function of the concentration of lithium in the organ (*C*_*i*_) divided by the partitioning coefficient *R*_*i*_ that characterizes the distribution of lithium between the plasma and the organ/tissue. The mass of lithium leaving the organ is often lower than entering due to the tissue to plasma equilibrium distribution ratio, resulting in lithium accumulation in that tissue. For all organs (except the kidneys), the rate of lithium clearance within the organ is negligible, and the only mode of lithium removal is through partitioning of lithium back from the tissue to the blood. Therefore, the general mass balance presented above in (1) can be modified with these described adjustments, resulting in the following.

**General compartment mass balance without clearance or absorption:**

(2) $$\begin{array}{c} \frac{d}{dt}\left(V_{i}C_{i}\right)=Q_{i}C_{\text{P}}-Q_{i}\frac{C_{i}}{R_{i}} \end{array}$$

Assuming that the volume of each compartment is constant, the time-dependent concentration of lithium in the general tissue compartment *i*  can be expressed as follows.

**Constant-volume compartment mass balance without clearance or absorption:**

(3) $$\frac{dC_{i}}{dt}\;=Q_{i}\left(C_{\text{P}}-\frac{C_{i}}{R_{i}}\right)\frac{1}{V_{i}}$$

This general form applies to the brain, the thyroid, and the bone; the differential equations characterizing lithium concentration in these compartments are described in (4), (5), and (6) below.

**Brain (brain compartment mass balance):**

(4) $$\frac{dC_{\text{Br}}}{dt}=Q_{\text{Br}}\left(C_{\text{P}}-\frac{C_{\text{Br}}}{R_{\text{Br}}}\right)\frac{1}{V_{\text{Br}}}$$

**Thyroid (thyroid compartment mass balance):**

(5) $$\frac{dC_{\text{T}}}{dt}=Q_{\text{T}}\left(C_{\text{P}}-\frac{C_{\text{T}}}{R_{\text{T}}}\right)\frac{1}{V_{\text{T}}}$$

**Bone (bone compartment mass balance):**

(6) $$\frac{dC_{\text{B}}}{dt}=Q_{\text{B}}\left(C_{\text{P}}-\frac{C_{\text{B}}}{R_{\text{B}}}\right)\frac{1}{V_{\text{B}}}$$

The connectivity between the uterus and the fetus, the clearance of lithium from the kidneys, the absorption of lithium into the gut, and the contribution of all tissues to the plasma concentration lead to additional terms in the differential equations for these compartments; further discussion is provided below for each compartment with a form modified from the general one provided above in (3).

**Uterus (uterus compartment mass balance):**

(7) $$\frac{dC_{\text{U}}}{dt}=\left(Q_{\text{U}}\left(C_{\text{P}}-\frac{C_{\text{U}}}{R_{\text{U}}}\right)+Q_{\text{F}}\left(\frac{C_{\text{F}}}{R_{\text{F}}}-\frac{C_{\text{U}}}{R_{\text{U}}}\right)\right)\frac{1}{V_{\text{U}}}$$

Blood flow to the uterus comes from both the plasma compartment (at flow rate *Q*_U_ and at the plasma lithium concentration *C*_P_) as well as from the fetus compartment (at flow rate *Q*_F_ and at the lithium concentration leaving the fetus, *C*_F_/*R*_F_), and so there are two inflow terms in the uterus mass balance, as can be seen in (7). Furthermore, the flow exiting the uterus is at a lithium concentration of *C*_U_/*R*_U_, and it exits at flow rate *Q*_F_ (to the fetus) and at flow rate *Q*_U_ (back to the plasma compartment), providing two outflow terms to the uterus compartment mass balance.

**Fetus (fetus compartment mass balance):**

(8) $$\frac{dC_{\text{F}}}{dt}=Q_{\text{F}}\left(\frac{C_{\text{U}}}{R_{\text{U}}}-\frac{C_{\text{F}}}{R_{\text{F}}}\right)\frac{1}{V_{\text{F}}}$$

Because the fetus is connected through the body only through the uterus, and not directly to the plasma compartment, the blood entering and leaving the fetus passes first through the uterus, as shown above in (8). The partition function for the fetus, *R*_F_, also describes the partitioning between the fetus compartment and the plasma.

**Kidneys (kidney compartment mass balance):**

(9) $$\frac{dC_{\text{K}}}{dt}=\left(Q_{\text{K}}\left(C_{\text{P}}-\frac{C_{\text{K}}}{R_{\text{K}}}\right)-kk\frac{C_{\text{K}}}{R_{\text{K}}}\right)\frac{1}{V_{\text{K}}}$$

In the kidney compartment mass balance (9), the term *kk* refers to the kidney clearance rate (Table 1). The term *kk*∗*C*_K_/*R*_K_  gives the rate at which lithium is cleared from the body through the kidneys.

**GI tract (GI tract compartment mass balance):**

(10) $$\frac{dC_{G}}{dt}=\left(Q_{G}\left(C_{\text{P}}-\frac{C_{\text{G}}}{R_{\text{G}}}\;\right)+G\left(t\right)\right)\frac{1}{V_{G}}$$

The parameter *G*(*t*) is the rate (mEq/minute) of lithium absorption into the blood through the GI tract. In this study, we assumed all lithium drug was delivered orally as a time-release capsule. This term is only nonzero during the release of lithium from an active drug dosing time period. Assuming that the time-release format of the drug delivery would result in a constant rate of absorption over the course of the drug dissolution time, the absorption term *G*(*t*) in (10) above was calculated as follows.

**Drug absorption term:**

(11) $$G\left(t\right)=\frac{m_{D}\left(\text{mEq}\;Li/\text{mg}\;\text{drug}\right)}{\Delta t}$$

In (11), *m*_*D*_ is the mass of the drug dose in mg, mEq  *Li*/mg  drug is the conversion factor for lithium active ingredient contained in each mg of drug, and Δ*t* is the time over which the constant rate absorption of the time-release capsule occurs. According to [3], peak serum concentrations occur 4–12 hours after dosing with controlled release lithium drug. Logically, this implies that the last of the lithium drug is delivered to the system 4–12 hours after the dose is administered. By utilizing the lower end of this range of drug absorption time, a “worst-case” scenario for peak tissue concentration with a time-release drug regimen will be obtained. Therefore, the default assumption for the duration of drug absorption during administration was to use Δ*t* = 4 hours in (11). The timing of the predicted peak in plasma lithium concentration in the model that corresponds to this *G*(*t*) is four hours, confirming that the peak concentration corresponds with the end of drug release and absorption in our model.

**Plasma (plasma compartment mass balance):**

(12) $$\begin{array}{cc} \frac{dC_{\text{P}}}{dt} & =[Q_{\text{G}}\frac{C_{\text{G}}}{R_{\text{G}}}+Q_{\text{K}}\frac{C_{\text{K}}}{R_{\text{K}}}+Q_{\text{B}}\frac{C_{\text{B}}}{R_{\text{B}}}+Q_{\text{U}}\frac{C_{\text{U}}}{R_{\text{U}}}+Q_{\text{T}}\frac{C_{\text{T}}}{R_{\text{T}}}\\ & \;\;+\;\;Q_{\text{Br}}\frac{C_{\text{Br}}}{R_{\text{Br}}}-\left(Q_{\text{G}}+Q_{\text{K}}+{Q_{\text{B}}+Q}_{\text{U}}+Q_{\text{T}}+Q_{\text{Br}}\right)C_{\text{P}}]\frac{1}{V_{\text{P}}} \end{array}$$

The mass balance on the plasma compartment includes the sum of all the outflows from and inflows to each of the individual compartments included in the model.

## 3. Results

All simulations were performed using the MATLAB software suite. In the medical literature, bodily lithium concentrations are reported in milliequivalents lithium/mL tissue volume (mEq/mL); the unit mEq is equivalent to a millimol. In this paper, we utilize the units mEq to report bodily concentrations to be consistent with the medical literature. Dosages of lithium drugs are reported in milligram of the total drug, which is lithium carbonate (Li_2_CO_3_) for the brand name drugs Eskolith and Lithobid. Therefore, for example, a 300 mg tablet contains 8 mEq lithium. 

### 3.1. Lithium Concentration Profiles for a Single 900 mg Dose of Lithium Drug (as Li_2_CO_3_)

**Figure 2:**
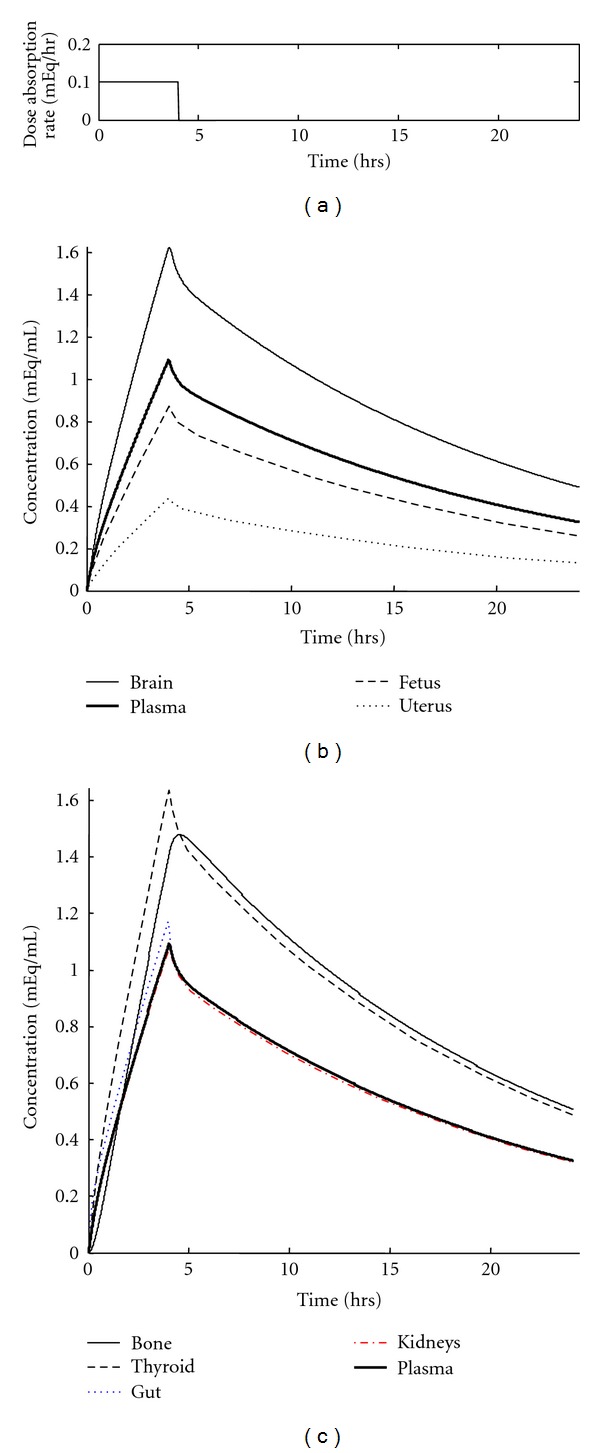
Concentration profiles in all physiological compartments resulting from a single, time-release 900 mg dosage of lithium drug. Initial lithium concentration in the body is 0 mEq/mL. (a) Profile of drug release pulse. (b) Lithium concentration time courses in the most important compartments for the study, with fetus labeled. (c) Lithium concentration profiles for less critical compartments.

A typical dosage regimen for an individual suffering from bipolar disorder is to administer 900 mg of lithium drug twice daily. The lithium concentration time course (in mEq Li/mL) resulting from a single 900 mg dose of lithium drug, delivered via two simultaneously administered 450 mg controlled release capsules, results in the concentration profiles shown in Figure 2. The organs shown in Figure 2(b) are important for the aim of this study, which was to provide better guidance for safe lithium dosing for fetal development; the concentration profiles shown in Figure 2(c) are other organs included in the model but less critical for the aim of this study. The drug release occurs over four hours, as described above, in conjunction with the mass-balance development for the GI Tract; after the last of the delivered drug has absorbed into the system, all concentrations decay due to loss of lithium through the urine via kidney clearance. Our simulation uses a flow rate of urine containing lithium of 20 mL/minute; this value is consistent with the standard accepted values of 20–40 mL/minute [3].

The concentration in each organ is based on the tissue to plasma equilibrium distribution ratio, *R*_*i*_. For example, the concentration in the uterus is less than half that of the plasma because the ratio for lithium partitioning into the uterus from the plasma (*R*_U_) is 0.4 [3].

These concentration profiles fit the known data. There is a peak in plasma lithium concentration at approximately 4 hrs, concurrent with the end of drug dissolution from the time-release capsule. The half-life of lithium can be approximated as the time it takes for the lithium in the plasma to decrease 50% from its maximum value. In our model, this half-life is approximately 12 hours, which is consistent with the reported serum half-life of lithium for pregnant women [4].

### 3.2. Lithium Concentration Profiles for Dosage Regimens Known to Cause Birth Defects

**Figure 3:**
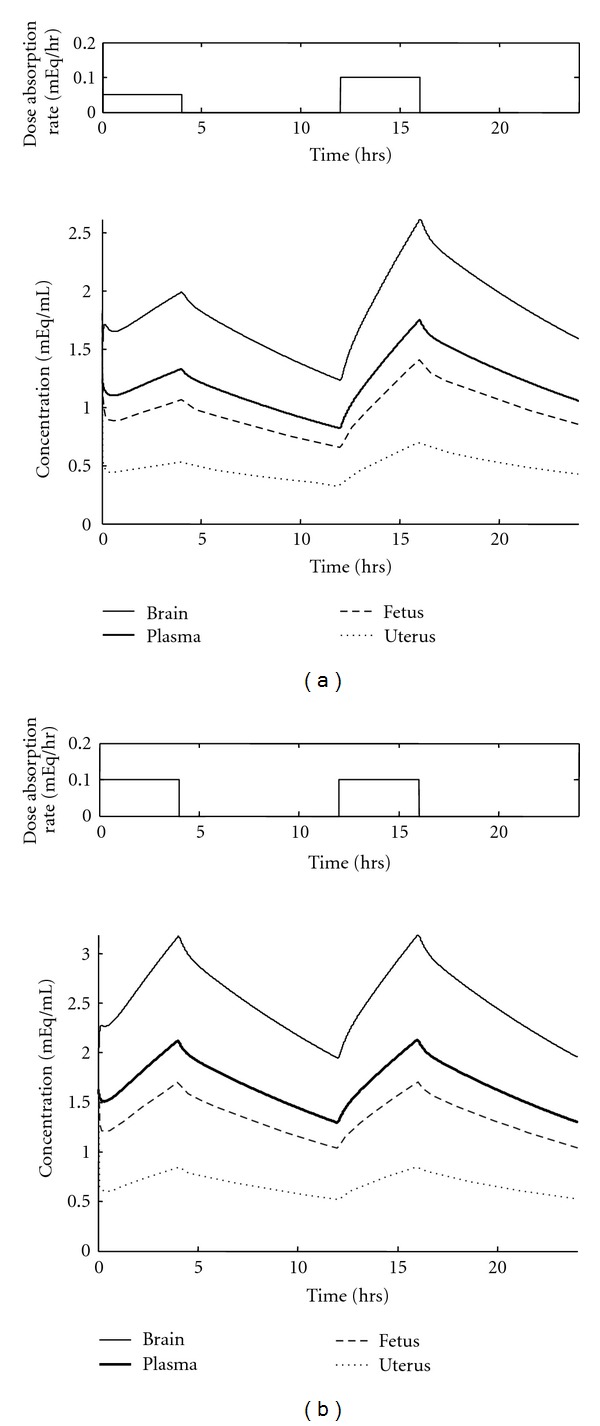
Terminal concentration profiles in selected physiological compartments for dosage regimens that are known to cause birth defects. In this case, lithium medication is administered twice daily and controlled-release tablets release lithium over 4 hours. A pulse function corresponding to the drug absorption is shown above each figure. (a) One dose of a 450 mg tablet (12 mEq lithium) with a subsequent 900 mg (24 mEq lithium) dose. (b) Two doses of a 900 mg tablet (24 mEq lithium).

Lithium is known to cause birth defects in infants; although there have been no clinical trials to ascertain the exact dosage that causes harm. There are, however, several reported cases of women who continued to take lithium during their pregnancy, gave birth to a baby with birth defects, and the doctors reported the dosage regimen. One-dosage regimen that has been documented to cause birth defects was two capsules daily, 450 mg in the morning followed by 900 mg in the evening [2]. There are numerous sources, including the International Register of Lithium Babies, that document birth defects as a result of treatment with lithium. However, the dosage regimens used are not reported or accessible. The standard dosage of two 900 mg doses daily was used to describe the dosage profiles for these cases. The predicted lithium concentrations over time associated with these dosing regimens are shown in Figure 3 for the brain, plasma, fetus, and the uterus.

For both dosage regimens, the figure shows the concentration profile after the patient has been taking the drug for a long period of time; this means that every two-dose cycle has the exact same profile, which will be referred to as the terminal profile. The first regimen with alternating dosage sizes resulted in a concentration profile of high and low peaks. The maximum fetus concentration associated with the 450 mg/900 mg regimen is 1.4 mEq/mL, with an average concentration of 0.97 mEq/mL. For the second regimen of two 900 mg doses daily, the peak fetus concentration is 1.7 mEq/mL and an average concentration of 1.3 mEq/mL. Both peaks and means were found using a terminal profile.

Birth defects could be caused by either the maximum concentration experienced by the fetus, or the average concentration of lithium in the fetus over the gestation period; the data presently available does not conclusively point to either of these as the primary cause of the birth deformations. While this information cannot be utilized to recommend a “safe” dosage regimen that can guarantee a defect-free fetus, there are two useful conclusions from these results.

The first and most useful output from this analysis is to provide, from the 450 mg/900 mg dosage regimen, the following concentrations which should not be exceeded during lithium treatment of a pregnant woman:

1. a *maximum* peak fetal lithium concentration,
2. an *average* fetal lithium concentration.

These standards can be used to identify lithium therapy regimens likely to exceed these known pathological concentrations, so that their administration to pregnant patients can be prevented.

The second output is the ability to now use the model to suggest dosage regimens that will result in fetal concentrations that fall significantly below the known pathological levels, while still maintain high enough concentrations to be effective for the mother; this is the topic of the next section.

### 3.3. Lithium Dosage Regimens Eliminated Based on Model Results

**Figure 4:**
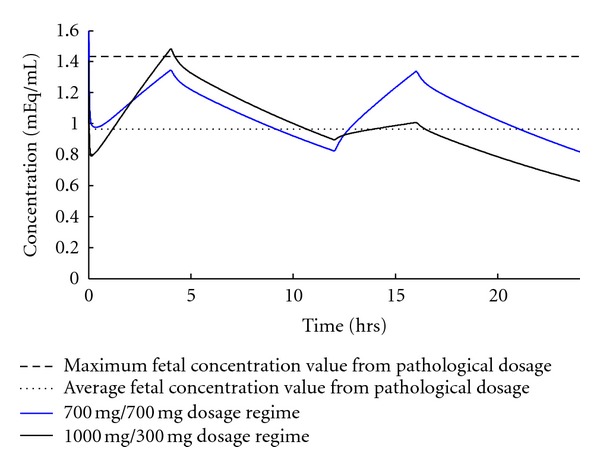
Model-predicted pathological dosage regimens. The maximum and average fetus concentrations from the 450/900 dosage regimen are plotted along with two new dosage regimens. A 300/1000 dosage regimen is shown in black and a 700/700 dosage regimen is shown in blue.

 The previously documented pathological cases are primarily useful in ruling out other potential dosage regimens. To this end, we selected two dosing regimens within the therapeutic dose range where the effect on the fetus is unknown. The first dosage regimen we modeled is two 700 mg doses, 12 hrs apart (denoted 700/700). The next dosage regimen is a 1000 mg dose followed by a 300 mg dose 12 hrs later. The results of these simulations are shown in Figure 4.

The 700/700 dosage regimen gives a higher average concentration than the pathological dosage, while maintaining a lower peak concentration. This is because the doses are more “spread out” across the day. The 1000/300 dosage regimen gives a higher peak than the pathological dosage, while maintaining a lower average. Neither of these regimens should be considered for pregnant women because they cross the pathological dosage either average or peak concentration and therefore have the potential to cause birth defects. The power behind this conclusion is that the model used clinical pathological data to eliminate dosing regimens that have an unknown/undocumented effect on a fetus.

### 3.4. Model-Suggested Reduced Risk Lithium Dosage Regimens

**Figure 5:**
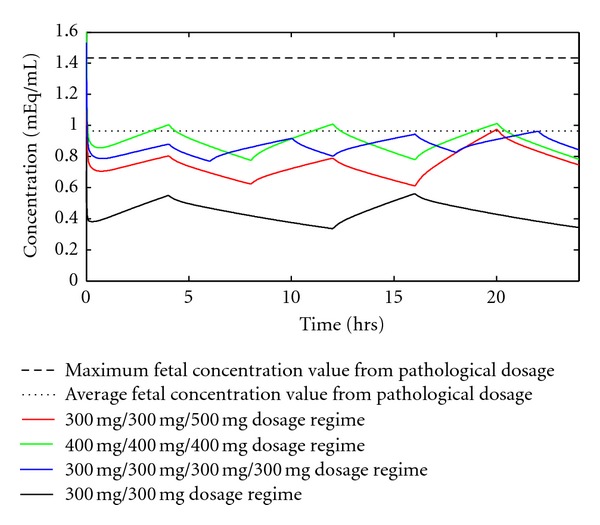
Model-predicted reduced risk dosage regimens. The maximum and average fetus concentrations from the 450/900 dosage regimen are plotted along with two new dosage regimens. The values for average and peak concentrations are listed in Table 2.

In order to find safer dosage regimens, we modeled several regimens to find ones with peak and average concentrations below the pathological dosage. Drug ingestion does not have to occur only twice daily, and the regimens we tested reflect this. However, we did not consider the effect of nonevenly spaced dosages which, due to its complexity and questionable clinical relevance, is beyond the scope of the current work. We modeled the following regimens: 300/300, 600/600, 300/300/500, 400/400/400, and 300/300/300/300 (all in mg). We included the 300/300 dosage regimen because this has been suggested as an average lowest effective dosage regimen [2]. Although this varies for each patient, it is a good starting value for minimum effective dose. The 300/300/500 dosage regimen simulates two low doses with breakfast and lunch and a slightly higher dosage with dinner to go through the night.  Figure 5 show the results of several of these simulations.  Table 2 shows the maximum and average concentration values of these dosage regimens.

All of the dosage regimens modeled show both a lower peak concentration and average concentration than the pathological dosage. We noticed several interesting aspects when evaluating these simulations. First, fewer doses per day give a higher peak concentration at constant daily dosage. For example, the 600/600 regimen (not shown) has a peak concentration of 1.141 mEq/mL. The 400/400/400 regimen, however, has a peak concentration of 1.019 mEq/mL. Second, higher peak concentrations do not necessarily mean a higher average concentration. The data from the three 1200 mg/day dosages (300/300/300/300, 400/400/400, and 600/600) show that predicting average fetus concentration from total dosage, number of doses, or peak fetus concentration can be difficult or nonintuitive.

In order to find a recommended maximum reduced risk regimen, we need to consider both the peak and average concentrations. All of the dosage regimens displayed in Figure 5 show average and peak concentrations below the pathological dosage; therefore, they are all acceptable from a quantitative viewpoint. The “best” recommended dosage regimens to reduce risk are the 300/300/300/300 and 400/400/400 regimens. The 300/300/300/300 regimen is the best quantitatively. The average concentration is only slightly less than the pathological dosage, but no peak goes above the pathological dosage average. The downfall of this dosage regimen, however, is that four doses per day may be difficult for patients to reasonably take. For compliant patients who are concerned with the welfare of the child and need high lithium dosages, the 300/300/300/300 dosage regimen is the best maximum regimen. For any other patient, a dosage regimen that allows for taking the drug with meals will be much more convenient and likely to succeed in a clinical setting. The best maximum regimen for most patients will therefore be the 400/400/400 dosage regimen. This regimen keeps the average concentration slightly lower than the 300/300/300/300 regimen, and the peaks in the concentration profile go slightly above the Pathological Dosage average concentration. However, since only the peaks are higher than the pathological dosage average concentration, there should still be reduced risk associated with this dosing regimen. It should be noted that this best dosage regimen was determined from the results of a simulation which has not been validated by animal or clinical trials; furthermore the treatment of patients deals with individuals. Because individuals respond to drugs differently and have many different medical histories, the optimum dose (and even the maximum safe dose) will vary with individual patients. The results of this study should therefore be taken as a guide to safely treat patients rather than universal canon.

## 4. Conclusion

This model has taken the first steps toward predicting the maximum acceptable lithium dosage regimen for pregnant bipolar women. Based on our simulation results and on clinical patient compliance, we recommend a maximum dosage regimen of three doses of 400 mg lithium evenly spaced over a 24-hour period. It is important to note that the maximum dosages recommended from the model are not necessarily nonpathological. These recommended dose regimens simply lower the average and peak values below concentrations known to be pathological. It is still important to take the lowest effective dosage of lithium to minimize the risk of birth defects. Hopefully, this paper has shed some light on what the maximum doses in a dosage regimen should look like to lower the risk of birth defects. Future research to improve the model could include determination of the fetal tissue to plasma lithium equilibrium ratio and collecting data on “safe” dosages of lithium that do not cause birth defects.

## Figures and Tables

**Table 1:** Model parameters.

Conversion factor for lithium content of drug dose	8/300	mEq (mmol) Li/mg dose	[3]

Bone mass	8	kg	[14]

Bone density	1.1	g/mL	[15]

Kidney clearance rate (*kk*)	20	mL/min	[3]

Physiological volumes of organ/tissue compartments			

Plasma (*V*_P_)	5200	mL	[16]
Bone (*V*_B_)	7273	mL	Calculated from bone mass and bone density
Kidney (*V*_K_)	280	mL	[16]
Uterus (*V*_U_)	1000	mL	[7]
Gastrointestinal (GI) tract (*V*_G_)	1650	mL	[16]
Thyroid (*V*_T_)	13	mL	[16]
Brain (*V*_Br_)	1450	mL	[16]
Fetus, 1st Trimester (*V*_F_)	150	mL	[7]

Blood flow to organs			

Bone (*Q*_B_)	272	mL/min	Calculated from bone perfusion and bone mass [14]
Kidney (*Q*_K_)	1240	mL/min	[16]
Uterus (*Q*_U_)	475	mL/min	[7]
GI Tract (*Q*_G_)	1100	mL/min	[16]
Thyroid (*Q*_T_)	60	mL/min	Modeled based on similar sized organs
Brain (*Q*_Br_)	700	mL/min	[16]
Fetus, 1st trimester (*Q*_F_)	300	mL/min	[7]

Tissue to plasma equilibrium distribution ratios for lithium			

Bone (*R*_B_)	1.5		[3]
Kidney (*R*_K_)	1		[3]
Uterus (*R*_U_)	0.4		[3]
GI Tract (*R*_G_)	1		[3]
Thyroid (*R*_T_)	1.5		[3]
Brain (*R*_Br_)	1.5		[3]
Fetus (*R*_F_)	0.8		Estimated; see rationale in text

**Table 2:** Average and maximum fetal concentrations (mEq/mL) for suggested dosage regimens compared with the pathological case. All dosages are over a 24 hour period.

Dosage regimen	Average fetal concentration (mEq/mL)	Maximum fetal concentration (mEq/mL)
450 mg/900 mg (pathological)	0.965	1.434
300 mg/300 mg/500 mg	0.796	1.021
300 mg/300 mg	0.419	0.570
300 mg/300 mg/300 mg/300 mg	0.867	0.960
400 mg/400 mg/400 mg	0.860	1.019
600 mg/600 mg	0.886	1.141

## References

[1] National Institute of Mental Health (2009). Bipolar Disorder. *NIH Publication*.

[2] Kozma C (2005). Neonatal toxicity and transient neurodevelopmental deficits following prenatal exposure to lithium: another clinical report and a review of the literature. *American Journal of Medical Genetics*.

[3] Lexi-Comp Online (2011). *Formulary and Drug Therapy Guide: Lithium*.

[4] Blake LD, Lucas DN, Aziz K, Castello-Cortes A, Robinson PN (2008). Lithium toxicity and the parturient: case report and literature review. *International Journal of Obstetric Anesthesia*.

[5] Bendz H, Schön S, Attman PO, Aurell M (2010). Renal failure occurs in chronic lithium treatment but is uncommon. *Kidney International*.

[6] Blackburn ST (2007). *Maternal, Fetal, & Neonatal Physiology: A Clinical Perspective*.

[7] Cunningham FG, Leveno KJ, Bloom S, Hauth JC, Rouse D, Spong C (2009). *Williams Obstetrics*.

[8] http://dailymed.nlm.nih.gov/dailymed/lookup.cfm?setid=ea4ece7f-e81f-48de-b262-577db5b6fe6c.

[9] Bischoff KB, Dedrick RL, Zaharko DS, Longstreth JA (1971). Methotrexate pharmacokinetics. *Journal of Pharmaceutical Sciences*.

[10] Timchalk C, Nolan RJ, Mendrala AL, Dittenber DA, Brzak KA, Mattsson JL (2002). A physiologically based pharmacokinetic and pharmacodynamic (PBPK/PD) model for the organophosphate insecticide chlorpyrifos in rats and humans. *Toxicological Sciences*.

[11] Tardif R, Charest-Tardif G, Brodeur J, Krishnan K (1997). Physiologically based pharmacokinetic modeling of a ternary mixture of alkyl benzenes in rats and humans. *Toxicology and Applied Pharmacology*.

[12] Jones HM, Gardner IB, Watson KJ (2009). Modelling and PBPK simulation in drug discovery. *AAPS Journal*.

[13] Bischoff KB, Dedrick RL, Zaharko DS (1970). Preliminary model for methotrexate pharmacokinetics. *Journal of Pharmaceutical Sciences*.

[14] Schoutens A, Arlet J, Gardeniers JWM, Hughes SPF (1993). *Bone Circulation and Vascularization in Normal and Pathlogical Conditions*.

[15] Weatherall DJ, Ledingham JGG, Warrell DA (1996). The skeletal system. *Oxford Textbook of Medicine*.

[16] Davies B, Morris T (1993). Physiological parameters in laboratory animals and humans. *Pharmaceutical Research*.

